# Dopingberichterstattung in den Medien: Subjektive Wahrnehmungen, Bewertungen und vermutete Effekte bei Spitzensportlern

**DOI:** 10.1007/s00103-026-04250-6

**Published:** 2026-06-02

**Authors:** Michael Schaffrath, Thorsten Schulz

**Affiliations:** 1 Arbeitsbereich für Medien und Kommunikation, Department Health and Sport Sciences, TUM School of Medicine and Health, Technische Universität München, Am Olympiacampus 11, 80809 München, Deutschland; 2 Lehrstuhl für Präventive Pädiatrie, Department Health and Sport Sciences, TUM School of Medicine and Health, Technische Universität München, München, Deutschland

**Keywords:** Doping, Spitzensportler, Sportjournalismus, Reziproke Effekte, Online-Befragung, Doping, Top athletes, Sports journalism, Reciprocal effects, Online survey

## Abstract

**Einleitung:**

Im Fokus der Dopingberichterstattung stehen fast immer nur Athleten. Doch rezipieren diese überhaupt die Berichterstattung über Doping? Wie wird die Dopingberichterstattung bewertet? Und welche Einflüsse können Dopingberichte auf Spitzensportler haben?

**Methode:**

2025 wurde eine Online-Befragung unter deutschen Kaderathleten durchgeführt. 349 Fragebögen wurden deskriptiv und inferenzstatistisch ausgewertet.

**Ergebnisse:**

85 % der Athleten rezipieren Dopingberichte, um zu erfahren, wie es zu einem Dopingfall kommt und wie Medien dann damit umgehen. 62 % der Befragten freuen sich, wenn Dopingsünder erwischt werden und die Medien darüber berichten. Enttäuschung stellt sich bei 60 % ein, weil Dopingberichte das Image der eigenen Sportart beschädigen können. 38 % der Athleten sind wütend darüber, dass die Medien fast ausschließlich Sportler kritisieren, aber keine anderen für Doping mitverantwortlichen Akteure. Einen Einfluss auf die mentale oder körperliche Leistungsfähigkeit können sich 15 % der Befragten vorstellen, wobei für einige Befragte eine höhere Trainingsintensität und größere Wettkampfmotivation denkbar sind, während andere Trainingsreduktion und Demotivation im Wettkampf vermuten. Die Variablen Alter und Geschlecht spielen im Antwortverhalten keine Rolle.

**Diskussion:**

Die Mehrheit der Athleten rezipiert die Dopingberichte aus einem Informationsbedürfnis heraus und viele Befragte befürworten die normative Kritik- und Kontrollfunktion der Medien. Dopingberichte werden zwar als imageschädigend für die eigene Sportart empfunden. Aber die Aufklärungsarbeit der Medien und die Demaskierung von Dopingsündern werden auch als ein wichtiger Beitrag zur Aufrechterhaltung eines fairen sportlichen Wettbewerbs gesehen.

**Zusatzmaterial online:**

Zusätzliche Informationen sind in der Online-Version dieses Artikels (10.1007/s00103-026-04250-6) enthalten.

## Abstract (en)

**Introduction:**

Media coverage of doping almost always focuses solely on athletes. But do they even read reports on doping? How do athletes evaluate doping coverage? And what impact can doping reports have on elite athletes?

**Method:**

In 2025, a quantitative online survey was conducted among German national team athletes. Using descriptive and inferential statistics, 349 questionnaires were analyzed.

**Results:**

Of the athletes, 85% read doping reports to learn how a doping case arises and how the media handles it. Of the respondents, 62% are pleased when doping offenders are caught and subsequently exposed through media coverage. Disappointment sets in for 60% because doping reports can damage the image of their own sport, and 38% of athletes expressed frustration with the media’s tendency to almost exclusively criticize athletes but not other groups who share responsibility for doping. Approximately 15% of respondents believe doping reports can influence mental or physical performance, with some respondents suggesting increased training intensity and greater motivation in competition, while others suspect reduced training and demotivation in competition. The variables of age and gender do not play a role in the response patterns.

**Discussion:**

The majority of athletes follow doping reports primarily out of a need for information and support the media’s role in providing normative criticism and control. While doping reports are perceived as damaging to the image of their own sport, the media’s educational efforts and the exposure of doping offenders are also seen as an important contribution to maintaining fair competition in sports.

## Einleitung

Bei Dopingskandalen geraten fast immer die Athleten in den medialen Fokus. Das liegt daran, dass sich Spitzensport grundsätzlich im Handeln der Athleten als den zentralen „Sozialfiguren“ manifestiert [1]. Sie müssen den Systemcode des Sports von Sieg und Niederlage umsetzen und sind dabei in öffentlich beobachtbaren Situationen präsent [1, 2]. „Dopingsünder, die man vorführen, zum Sprechen bringen oder in ihrem Schweigen zeigen kann, lassen sich von Zuschauern relativ leicht wahrnehmen. Die Einzelfallbehandlung des Sports in Sachen Doping passt in die Selbstbezüglichkeit der Medien hinein“ [3]. Das liegt daran, dass Doping eine Reihe von Nachrichtenfaktoren erfüllt, die die journalistische Selektion und mediale Präsentation steuern. Dies sind vor allem „Personalisierung“ und „Skandalisierung“ [4–7], wie verschiedene Inhaltsanalysen von Medienberichten belegt haben [8–10].

Personalisierung: „Nichts ist für die Medien informativer als ein Ereignis, das an einzelnen Personen in einer kompakten und leicht nachvollziehbaren Weise sichtbar gemacht werden kann“ [3]. Die Personalisierung zielt darauf ab, den Dopingsünder als Allein- oder als Hauptverantwortlichen für das aufgedeckte Vergehen in den Fokus des öffentlichen Interesses zu rücken [4, 6]. Die journalistische Einzelfallbetrachtung hat die Funktion, „die strukturelle Kopplung zwischen Massenmedien und Spitzensport abzusichern und als noch nicht gefährdet auszuweisen. Würden die Medien Doping nicht an einzelnen Personen festmachen, sondern als einen nicht mehr aufzuhaltenden, strukturell erzeugten Flächenbrand behandeln, könnten sie den Sport in eigener Sache nicht mehr nutzen“ [7].

Skandalisierung: Doping konterkariert konstitutive Maximen des Sports, wie etwa den offenen Wettkampfausgang oder die Chancengleichheit der Athleten [3]. Dopingsünder demoralisieren ethische Prinzipien und provozieren Irritationen sowie Erwartungsenttäuschungen in der Öffentlichkeit, ähnlich wie bestechliche Politiker oder korrupte Beamte [3, 5]. Doping ist ein Normverstoß. Und ein Normverstoß ist das „konstitutive Element jedes Skandals“ [11]. Dopingvergehen gehören damit zu den „Skandalen par excellence“ [11].

Trotz der personalisierten Skandalisierung einzelner Dopingsünder haben die Soziologen Bette und Schimank in diversen Publikationen darauf hingewiesen, dass es sich bei Doping um einen „Konstellationseffekt“ handelt, der in den „Strukturdynamiken“ des modernen Spitzensports angelegt ist und „der durch eine Vielzahl von Akteuren erzeugt wird“, ohne dass diese immer ihre eigene Mitverantwortung erkennen [3, 12, 13].

Die wissenschaftliche Auseinandersetzung mit dem Thema Dopingberichterstattung hat in den vergangenen 20 Jahren zugenommen [z. B. 4, 5, 14–17]. Empirisch wurden dabei in erster Linie Inhaltsanalysen zur Berichterstattung durchgeführt [4, 5]. Außerdem liegen einige Befragungen von Sportjournalisten [4, 5, 18–21] sowie eine Umfrage von Trainern [22] vor.

Befragungen von Spitzensportlern zum Thema Doping gibt es bisher wenige [23–32]. Überdies sind diese Befragungen kaum miteinander zu vergleichen. Das liegt an divergierenden Methoden (oft Tiefeninterviews oder halbstrukturierte Leitfadengespräche und selten quantitative Befragungen), an stark variierenden Stichprobengrößen (zwischen 5 und 468 Befragten), an unterschiedlichen Altersgruppen der Teilnehmer (12 bis 46 Jahre) oder an verschiedenen Herkunftsländern mit unterschiedlichen Sportkulturen (z. B. Australien, China, Uganda und USA). Noch gravierender erscheint, dass Athleten aus verschiedenen Sportarten befragt wurden, in denen Doping unterschiedlich verbreitet ist (von Badminton über Cricket, Gewichtheben, Handball, Fußball und Radsport bis Schwimmen und Wrestling). Zusätzlich zur Problematik der Unvergleichbarkeit der Studien zeigt sich, dass lediglich in 4 Untersuchungen rudimentäre Ergebnisse zur Dopingberichterstattung vorliegen [25–28]. Im Wesentlichen geht es in den Studien nur um Kritik an der Dopingberichterstattung. So wird von einzelnen Athleten moniert, dass Medien über Doping „ausschließlich negativ“ berichten [25], dass Medien bei der Zahl der Dopingfälle oft „übertreiben“ [28] oder dass Medien Sportler im Kontext von Doping als „Dummköpfe“ porträtieren [26].

Insgesamt kann der Forschungsstand quantitativ (wenige Studien, geringe Fallzahlen) und qualitativ (divergierende inhaltliche Schwerpunkte und Dopingberichterstattung nur als Randaspekt) als defizitär eingestuft werden. Daher sollte eine quantitative Befragung mit einer möglichst großen Stichprobe und einer inhaltlichen Fokussierung auf die Wahrnehmung und Bewertung der Dopingberichterstattung sowie auf die potenzielle Beeinflussung durch Dopingberichte entwickelt werden.

Als theoretische Rahmung der empirischen Studie dient der kommunikationswissenschaftliche Ansatz „reziproker Effekte“. Zentraler Ausgangspunkt ist das Konzept der „Wahrnehmung“. Wahrnehmung kann allgemein als komplexer kognitiver Prozess der Informationsgewinnung beschrieben werden, der es ermöglicht, die durch Sinnesorgane aufgenommenen Reize zu verarbeiten und zu interpretieren [33]. „Das Wahrgenommene wird von uns gefiltert, strukturiert sowie in unsere bisherigen Erfahrungen integriert und damit gedeutet. … Wir interpretieren das Gesehene auf der Grundlage unseres Vorwissens sowie gefärbt durch unsere persönlichen und kulturellen Wertvorstellungen, Einstellungen und Interessen“ [34]. Wahrnehmung erfolgt also subjektiv und selektiv. Und das dürfte bezogen auf den „Reiz“ Dopingberichterstattung genauso sein. Die Kommunikationswissenschaft geht davon aus, dass kommunikative Botschaften nur Wirkungen entfalten können, wenn sie von den Rezipienten vorher wahrgenommen werden [35]. Deshalb ist zunächst zu fragen, ob die Dopingberichterstattung überhaupt von den Spitzensportlern wahrgenommen wird, denn nur dann kann diese auch bewertet werden und gegebenenfalls Wirkungen entfalten.

Der Begriff „reziprok“ meint, dass in diesem Ansatz nicht von einem linearen Wirkungsmodell ausgegangen wird, sondern von wechselseitigen Effekten [36]. „Reziproke Effekte“ sind Wirkungen auf alle namentlich genannten Personen, über die Medien berichten. Sie können aber auch bei Mitgliedern von Organisationen auftreten, die nicht explizit genannt, aber medial mitthematisiert werden [37–39]. In der hier vorgelegten Studie ging es nicht um „reziproke Effekte“ der Dopingberichterstattung auf namentlich genannte Dopingsünder, sondern um die Wahrnehmung und die Wirkungen von Dopingberichten auf Athleten, die als Mitglieder von Verbänden und Vereinen oder als Ausübende einer Sportart, die in der medialen Berichterstattung mit Doping in Verbindung gebracht wird, betroffen sein können. Theoretisch vorstellbar sind auch Wirkungen bezüglich der physischen und mentalen Leistungsfähigkeit. Letztere meint die Kompetenz von Athleten, ihr Leistungspotenzial auch unter extremem Wettkampfdruck, bei Rückschlägen oder unter großer Belastung konstant abzurufen.

In dem Ansatz der reziproken Effekte werden 4 Wahrnehmungsdimensionen unterschieden [37, 39], die am Beispiel Dopingberichterstattung kurz erläutert werden sollen:

1. *Selbstwahrnehmung*: Spitzensportler machen sich ein Bild davon, ob der in den Medien dargestellte Dopingfall überhaupt vorliegt, ob die eigene Sportart involviert ist und inwieweit man selbst unmittelbar oder mittelbar von diesem Dopingskandal betroffen sein könnte.
2. *Berichtswahrnehmung*: Spitzensportler bilden sich anhand der Berichterstattung über Doping allgemein oder über einen konkreten Dopingfall eine Meinung darüber, was und wie die Medien berichten, wobei im Mittelpunkt der Meinungsbildung die Auswahl, Darstellung und Bewertung der Fakten stehen.
3. *Wirkungsvermutungen*: Spitzensportler überlegen sich, wie Dopingberichterstattung von anderen Personen, wie z. B. von Familie, Freunden, Teamkollegen, Sponsoren oder der sportinteressierten Bevölkerung, aufgenommen wird.
4. *Wirkungserfahrungen*: Dabei geht es um direkte Effekte der Dopingberichterstattung, die bei Spitzensportlern auftreten können. Das mögliche Spektrum reicht von der Verärgerung über präjudizierende und Gerüchte kolportierende Dopingberichte bis hin zu möglichen Einflüssen auf das Trainings- und Wettkampfverhalten der Athleten.

Außerdem sind bei „reziproken Effekten“ noch 3 Wirkungsdimensionen zu unterscheiden [33], die hier ebenfalls am Beispiel Dopingberichterstattung kurz exemplifiziert werden sollen:

1. *Kognitionen:* Dabei kann es um das durch Dopingberichte hervorgerufene Denken und Wissen zu einem Dopingskandal in der eigenen sowie in anderen Sportarten gehen bis zu Kenntnissen über die Motive anderer Athleten, sich zu dopen.
2. *Emotionen:* Hier handelt es sich um die durch Dopingberichte ausgelösten Empfindungen bei Spitzensportlern. Denkbar wären z. B. der Ärger über falsche Dopingberichte oder die Enttäuschung über dopende Teamkollegen, weil diese die eigene Sportart diskreditieren.
3. *Verhaltensweisen:* Damit sind direkt beobachtbare Effekte gemeint, die auch als Wechselwirkungen zwischen den 3 Wirkungsdimensionen auftreten können. Vorstellbar sind z. B. die Verweigerung von Interviews bis hin zu einer Veränderung des Trainings- und Wettkampfverhaltens.

Die auf Heldenkreierung angelegte Sportberichterstattung im Allgemeinen wird bereits bei mutmaßlichen Dopinggerüchten und sicher bei tatsächlichen Dopingskandalen zu einer auf Heldendemontage angelegten Dopingberichterstattung im Speziellen [3, 12]. „They [die Medien, die Autoren] go on about the importance of winning but then they picture you like some kind of cheating scum if you are found to be on something“ [26]. Diesen Vorwurf äußerte ein Athlet in einer internationalen Studie, bei der 18 Sportler mittels Leitfadengespräche interviewt wurden [26]. Handelt es sich hierbei um eine Einzelmeinung? Oder grundlegender gefragt: Wie nehmen Spitzensportler die Dopingberichterstattung wahr? Wie bewerten Spitzensportler die Dopingberichterstattung? Welche möglichen Wirkungen kann Dopingberichterstattung bei Spitzensportlern auslösen? Dies sind die 3 zentralen Forschungsfragen, denen im Folgenden nachgegangen wird.

## Methode

Ein Mixed-Methods-Design diente als Basis für die Studie. Da bisher nur wenige empirische Ergebnisse zu Wahrnehmung und Bewertung sowie keine Befunde zu möglichen Einflüssen der Dopingberichterstattung vorlagen, wurden zunächst qualitative Leitfadeninterviews mit Spitzensportlern durchgeführt. Um ein möglichst breites Spektrum an Einschätzungen zu erhalten, wurden die Interviewpartner aus unterschiedlichen Sportarten ausgewählt. Es waren Sportarten vertreten, in denen Doping häufiger und auch weniger häufig vorkommt, und zwar kategorisiert nach der Doping-Risikogruppeneinteilung der Nationale Anti Doping Agentur (NADA). Es sollten Einzel- und Mannschaftssportarten vertreten sein sowie Männer und Frauen. Die Auswahl der Athleten erfolgte auf Empfehlungen des Deutschen Olympischen Sportbunds (DOSB), von Athleten Deutschland oder über persönliche Kontakte.

Es wurden 10 männliche und 6 weibliche Athleten interviewt, die aus 16 verschiedenen Sportarten stammten: Radsport, Leichtathletik, Schwimmen, Ski Alpin, Nordische Kombination, Turnen, Kanu, Fechten, Judo, Rennrodeln, Rhythmische Sportgymnastik, Bogenschießen als Einzelsportart sowie Fußball, Handball, Hockey, Wasserball als Mannschaftssportarten. Die Interviews fanden als Vor-Ort-Leitfadengespräche statt und dauerten zwischen 30 min und 50 min. Die Gespräche wurden transkribiert und bildeten die Grundlage für die Entwicklung der quantitativen Online-Befragung. Weitere Anhaltspunkte für die Konstruktion des Fragebogens ergaben sich aus der Literatur zum Gegenstandsbereich [2, 3, 12–15] sowie aus den wenigen Athletenbefragungen, in denen die Dopingberichterstattung zumindest eine rudimentäre Rolle spielte [25–28]. Schließlich wurden auch Fragen aus den Vorgängerstudien mit Trainern und Journalisten übernommen [4, 22], um für weitergehende Auswertungen Vergleiche zwischen diesen 3 Akteursgruppen ziehen zu können.

Die quantitative Befragung wurde online durchgeführt, da diese Form der Datenerhebung „bei moralisch aufgeladenen Untersuchungsgegenständen“, wie es beim Thema Doping der Fall ist, „durch die Anonymität der Befragungssituation sogar validere Daten als die übrigen Varianten produzieren kann“ [40]. Es wurden 2 Pretests mit Studierenden und Mitarbeitern der Sportwissenschaft (*n* = 10) und danach mit Mitarbeitern vom DOSB, Athleten Deutschland, dem Olympiastützpunkt Bayern (OSP Bayern) und der Nationalen Anti Doping Agentur Deutschland (NADA; *n* = 6) durchgeführt.

Nach den Pretests umfasste der Fragebogen noch 32 Fragen mit 162 Items. Aus datenschutzrechtlichen Gründen wurde der Fragebogen über die 4 Kooperationspartner an die Athleten verschickt. Da manche Kader-Athleten bei verschiedenen Organisationen gelistet sind, ließen sich Mehrfach-Einladungen nicht verhindern. Der Versand erfolgte zunächst vom DOSB an 2703 Athleten (29.04.25), dann von Athleten Deutschland an seine 758 Mitglieder (06.05.25), dann vom OSP Bayern an 888 Sportler (22.05.25) und schließlich von der NADA an 114 Athleten (26.05.25). Der DOSB verschickte noch 2 Reminder, Athleten Deutschland und die NADA noch je einen. Mit diesem Vorgehen konnten Mehrfach-Teilnahmen nicht komplett ausgeschlossen werden. Aufgrund der von den 4 Kooperationspartnern als gering eingeschätzten Motivation, den Bogen 2‑mal auszufüllen, wurde diese Gefahr als marginal eingestuft.

Die Datenerhebung erfolgte im Zeitraum vom 29.04.2025 bis zum 10.07.2025. Insgesamt haben 411 Sportler den Umfragelink geöffnet. 365 Sportler haben die Umfrage gestartet und mindestens 2 Fragen beantwortet. Nach der Datenbereinigung konnten 349 Fragebögen in die Auswertung eingeschlossen werden. 236 Athleten haben die Umfrage beendet. Die über Unipark erhobenen Daten wurden exportiert, wobei die geschlossenen Fragen mit dem Statistikprogramm IBM SPSS Statistics Version 30,0 auf Gruppenunterschiede (Kreuztabelle mit Chi^2^-Test) und Spearman-Rangkorrelation ausgewertet wurden. Signifikante Unterschiede wurden auf dem 0,05-Signifikanzniveau berechnet.

## Ergebnisse

Die Stichprobe ist zwar nicht repräsentativ, weist aber angemessene Verteilungen nach Geschlecht, Alter und Bildungsgrad sowie bezüglich der Einteilung nach Doping-Risikogruppen der NADA auf (siehe Onlinematerial: Stichprobenstatistik).

### Wahrnehmung der Dopingberichterstattung

**Abb. 1:**
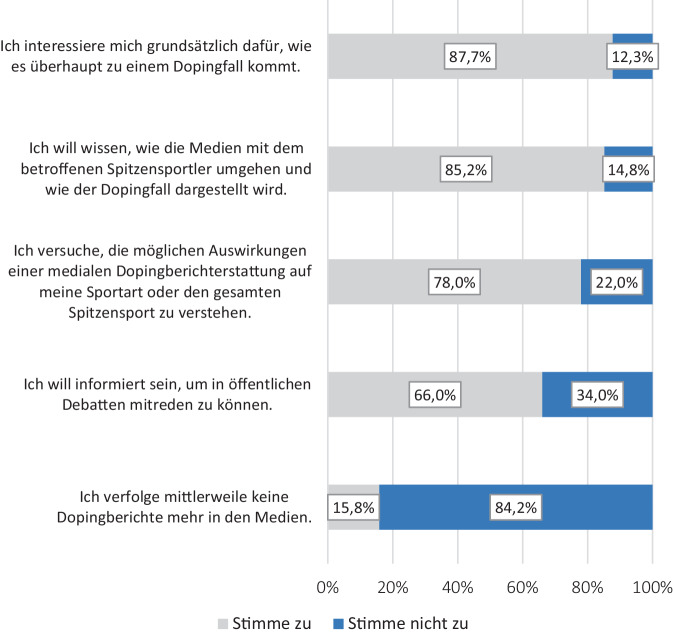
Gründe für die Rezeption der medialen Dopingberichterstattung, *n* = 236

Bezogen auf die Frage, ob Athleten die Dopingberichterstattung wahrnehmen, gaben 84,2 % an, dies zu tun. 15,8 % der Befragten verfolgen mittlerweile keine Dopingberichte mehr in den Medien (Abb. 1). Bei der Nachfrage, warum man keine Dopingberichte rezipiere, stimmten 63,0 % der Aussage zu, „weil ich prinzipiell kein Interesse an dem Thema Doping habe“, und 48,1 % meinten, „weil ich unzufrieden oder genervt bin, wie die Medien über einen Dopingfall berichten“.

87,7 % der Befragten verfolgen die Dopingberichterstattung, weil sie sich „grundsätzlich dafür interessieren, wie es überhaupt zu einem Dopingfall kommt“, 85,2 %, „weil sie wissen wollen, wie die Medien mit dem betroffenen Spitzensportler umgehen und wie der Dopingfall dargestellt wird“, 78,0 %, „um die möglichen Auswirkungen einer medialen Dopingberichterstattung auf meine Sportart oder den gesamten Spitzensport zu verstehen“, oder 66,0 %, „um in öffentlichen Debatten mitreden zu können“ (Abb. 1).

Ergänzend durchgeführte statistische Tests (Chi-Quadrat-Test und Fisher-Test) zeigen, dass die Variablen Geschlecht und Alter keinen signifikanten Einfluss auf das Antwortverhalten haben.

### Bewertung der Dopingberichterstattung

Die Dopingberichterstattung wird von vielen Athleten kritisch gesehen. So monieren 72,7 % der Befragten, dass die mediale Dopingberichterstattung „häufig spekulativ“ verlaufe. 75,1 % meinen, dass die Medien „zu schnell vorverurteilen“.

**Abb. 2:**
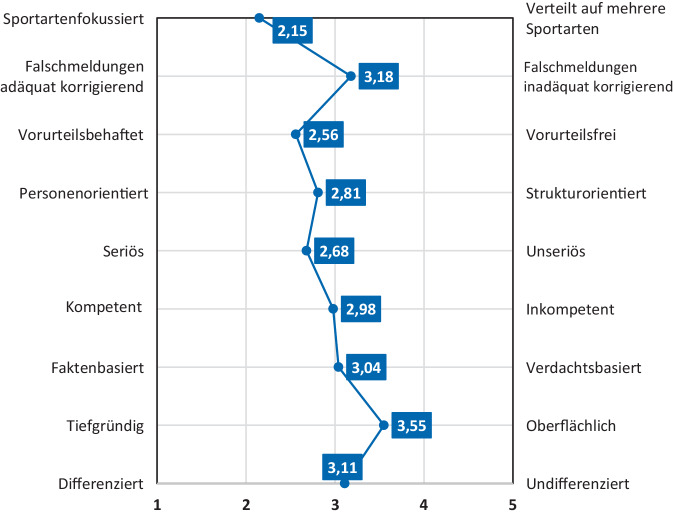
Semantisches Differenzial zur Bewertung der Dopingberichterstattung, *n* = 263

Neben diesen Einzelkritikpunkten sollten die Befragten anhand eines semantischen Differenzials die Dopingberichterstattung bewerten (Abb. 2). Es zeigt sich, dass nach Meinung der Athleten die Medien sich beim Thema Doping nur auf wenige ausgewählte Sportarten konzentrieren. Außerdem bewerten die Befragten die Dopingberichterstattung als eher „oberflächlich“, tendenziell „undifferenziert“ und auch eher „vorurteilsbehaftet“.

### Emotionale Effekte durch Dopingberichte

**Abb. 3:**
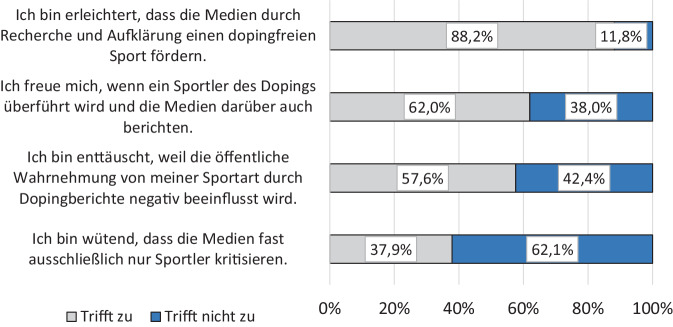
Emotionale Wirkungen durch Dopingberichterstattung, *n* = 161–193

Die Bewertung emotionaler Effekte, die durch Dopingberichte hervorgerufen werden, fallen unterschiedlich aus. 88,2 % der Befragten geben an, erleichtert zu sein, dass die Medien durch Recherchen und Aufklärungsarbeit einen Beitrag zu einem dopingfreien Sport liefern. 62,0 % der Athleten freuen sich, wenn dopende Sportler überführt werden und dies dann von den Medien öffentlich gemacht wird. Gleichzeitig sind 57,6 % der Athleten enttäuscht, dass die Berichterstattung über Doping die öffentliche Wahrnehmung der eigenen Sportart negativ beeinflusst. 37,9 % der Befragten geben an, sogar Wut darüber zu empfinden, dass Medien fast ausschließlich die Sportler kritisieren, wenn es um Doping geht (Abb. 3).

Auch bei dieser Frage besitzen die Variablen „Geschlecht“ oder „Alter“ keinen signifikanten Einfluss auf das Antwortverhalten bis auf eine Ausnahme. Bei der Wut über die ausschließliche Kritik an Sportlern zeigt sich ein signifikanter Unterschied zwischen Männern und Frauen. Von den männlichen Befragten stimmen 28,6 % dieser Aussage zu, von den weiblichen sind es 43,9 %. Der Chi-Quadrat-Test ergab einen statistisch signifikanten Zusammenhang zwischen Geschlecht und Zustimmung zu dieser Aussage (χ^2^(1) = 4,03, *p* = 0,045). Auch der exakte Fisher-Test bestätigte diesen Befund (*p* = 0,050; 2‑seitig). Die Effektstärke nach Cramérs V betrug 0,159, was einem kleinen bis mittleren Zusammenhang entspricht.

### Vermutete Wirkungen auf die mentale und körperliche Leistungsfähigkeit durch Dopingberichte

**Abb. 4:**
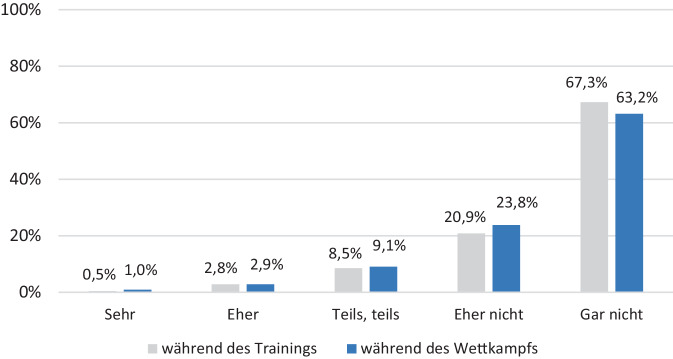
Einfluss von Dopingberichterstattung auf die mentale Leistungsfähigkeit, *n* = 209–211

**Abb. 5:**
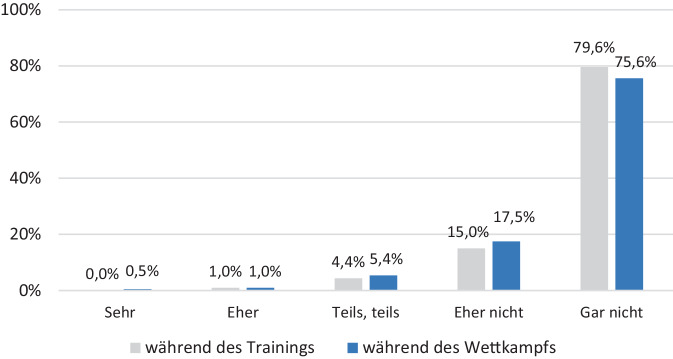
Einfluss von Dopingberichterstattung auf die körperliche Leistungsfähigkeit, *n* = 205–206

Die Mehrheit der Spitzensportler glaubt nicht daran, dass sie sich davon beeinflussen lässt, wenn Medien über Dopingfälle in ihrer Sportart berichten. Rund 88 % der Befragten meinen, dass Dopingberichte ihre mentale Leistungsfähigkeit „eher nicht“ oder „gar nicht“ beeinflussen können – weder im Training (88,2 %) noch im Wettkampf (87,0 %; Abb. 4). Ähnlich verhält es sich mit potenziellen Einflüssen auf die körperliche Leistungsfähigkeit. Hier geben auch fast alle Athleten an, dass sie sich von Dopingberichten „eher nicht“ oder „gar nicht“ beeinflussen lassen würden – weder im Training (84,6 %) noch im Wettkampf (93,1 %; Abb. 5).

Rund 12 % der Befragten geben an, dass Dopingberichte über die eigene Sportart ihre mentale Leistungsfähigkeit „teils, teils“, „eher“ oder „sehr“ während des Trainings und während des Wettkampfs beeinflussen würden. Hinsichtlich der körperlichen Leistungsfähigkeit in Training und Wettkampf machen rund 5 % der Athleten entsprechende Angaben. Die Variablen „Geschlecht“ und „Alter“ haben keinen signifikanten Einfluss auf das Antwortverhalten.

### Vermutete Wirkungen auf das sportliche Verhalten durch Dopingberichte

Ähnlich wie bei den Ergebnissen bzgl. der mentalen sowie der körperlichen Leistungsfähigkeit geben die meisten Befragten an, dass Dopingberichte in der eigenen Sportart keinen Einfluss auf das Verhalten im Training (84,5 %) und im Wettkampf (82,9 %) haben würden (Tab. 1).

**Tab. 1:** Einfluss der Dopingberichterstattung auf sportliches Verhalten, *n* = 205–206, *Inwieweit beeinflussen Dopingberichte in deiner Sportart dein Verhalten, bezüglich des Trainings und des Wettkampfs?*

Sport-Setting	Richtung der Beeinflussung		
Training	Eher positiv (trainingsverstärkend)	Eher negativ (trainingsreduzierend)	Gar nicht
	8,3 %	7,2 %	84,5 %
Wettkampf	Eher positiv (motivierend)	Eher negativ (demotivierend)	Gar nicht
	5,9 %	11,2 %	82,9 %

Von den jeweils rund 15 % der Sportler, die eine Beeinflussung durch Dopingberichterstattung für möglich halten, werden sowohl positive als auch negative Effekte angenommen. So geben 8,3 % der Befragten an, dass Berichte über Doping in der eigenen Sportart bei ihnen einen trainingsverstärkenden Effekt auslösen könnten. 5,9 % der Athleten halten eine zusätzliche Motivation im Wettkampf bei sich für denkbar. Demgegenüber meinen 7,2 % bzw. 11,2 % der Befragten, dass Dopingberichte bei ihnen eher eine Trainingsreduktion bzw. eine Demotivation im Wettkampf auslösen würden. Chi-Quadrat-Tests zeigen keinerlei signifikante Einflüsse der Variablen Geschlecht und Alter auf das Antwortverhalten.

## Diskussion

Die Studie zeigt, dass die Mehrheit der Befragten die Dopingberichterstattung wahrnimmt. Dies korrespondiert mit Ergebnissen aus vorherigen Untersuchungen [26, 28]. In früheren Studien wurde belegt, dass Spitzensportler den Einfluss der Medien auf die öffentliche Meinung sowie auf das Image der Sportler wahrnehmen [41]. Das scheint im Kontext von Doping ähnlich zu sein [42]. Wahrscheinlich deshalb wollen die meisten Athleten auch wissen, was und wie die Medien über einen Dopingfall berichten, welche Auswirkungen die Berichterstattung haben kann und ob bzw. inwieweit sie selbst mittelbar oder unmittelbar von den Dopingberichten betroffen sein könnten. Außerdem ist anzunehmen, dass die persönliche Betroffenheit der Athleten wächst, wenn über Doping in der eigenen Sportart berichtet wird. Wie die Ergebnisse zeigen, wollen die meisten Befragten informiert sein, um im öffentlichen Diskurs mitreden zu können.

Neben der kognitiven Dimension des Informiert-sein-Wollens können Dopingberichte auch emotionale Wirkungen evozieren. So konzediert die Mehrheit der Befragten z. B. Erleichterung darüber, dass Medien durch Recherchen und Berichterstattung einen dopingfreien Sport fördern, und empfindet Freude, wenn Sportler des Dopings überführt werden und die Medien dies öffentlich machen. Dies legt die Vermutung nahe, dass viele Athleten die normative Kritik- und Kontrollfunktion der Medien nicht nur akzeptieren, sondern deren Erfüllung befürworten, weil Doping die Chancen auf einen fairen Wettbewerb konterkariert und Dopingsünder die eigenen Siegchancen reduzieren. Die Mehrheit der Befragten befürchtet trotzdem, dass Dopingberichte die öffentliche Wahrnehmung der eigenen Sportart negativ beeinflussen können. Hier kann jedoch vermutet werden, dass eher das Dopen als imageschädigend empfunden wird und weniger die Berichterstattung darüber. In früheren internationalen Studien wurde ermittelt, dass vereinzelte Spitzensportler monieren, dass man die Schuld für Doping ausschließlich einzelnen Athleten zuschreibt und dies unter Ausblendung der Mitverantwortung anderer Umfeldakteure wie Trainer oder Funktionäre [23, 26]. In dieser Studie wurde konkret danach gefragt, ob die mediale Fokussierung auf die Athleten sogar Wut auslösen könnte. Bei einem Drittel der Befragten ist das der Fall, die Mehrheit empfindet aber so intensiv nicht.

Bezüglich der Bewertung der Dopingberichterstattung gab es in vorherigen Studien nur wenige Hinweise, die bereits im Forschungsstand skizziert worden sind [25, 26, 28]. In dieser Studie wurde nach weiteren sowie konkreteren Bewertungen gefragt. Rund 3 Viertel der Befragten artikulieren Vorwürfe, wie etwa, dass die Dopingberichterstattung oft „zu spekulativ“ und „zu präjudizierend“ verlaufe. Daneben zeigen die Einschätzungen beim semantischen Differenzial, dass auch die mediale Konzentration auf bestimmte Sportarten wahrgenommen wird, was frühere Inhaltsanalysen schon bestätigten [10, 43].

Verschiedene Autoren beschreiben Einflüsse von negativer Sportberichterstattung auf die mentale Gesundheit und auf das sportliche Leistungsvermögen von Athleten [44, 45]. So stellt Zhang fest, „the negative reports of various media have increasingly become an important factor affecting athletes’ competition attention and performance“ [45]. Davon ausgehend wurde in dieser Studie gefragt, ob auch Dopingberichte einen Einfluss auf die mentale oder körperliche Leistungsfähigkeit von Sportlern besitzen können. Die überwiegende Mehrheit der Athleten schließt das für sich selbst aus. Nur eine Minderheit kann sich vorstellen, dass Dopingberichte über die eigene Sportart sie im Training und Wettkampf beschäftigen.

Ähnlich verhält es sich, wenn gefragt wird, ob Dopingberichte das sportliche Verhalten der Athleten beeinflussen könnten. Auch hier negieren fast alle Befragten einen Einfluss auf ihr Verhalten im Training und im Wettkampf. Gleichwohl räumen 15 % der Befragten einen Einfluss auf ihr Verhalten ein. Dabei werden 2 Wirkungsrichtungen erkennbar. Manche Befragte geben an, durch Dopingberichte mehr zu trainieren und motivierter im Wettkampf zu sein. Dies legt die Vermutung nahe, dass diese Sportler zeigen wollen, dass man auch ohne verbotene Substanzen erfolgreich sein kann. Andere Befragte konstatieren, dass Dopingberichte bei ihnen eher zu Trainingsreduktion und Demotivation führen könnten. Dahinter steckt mutmaßlich die Befürchtung, dass man den durch das Dopen anderer Athleten erzeugten Vorteil letztlich nicht durch intensiveres Training und höhere Wettkampfmotivation kompensieren könne.

### Limitationen und Stärken

Die Stichprobe ist zwar nicht repräsentativ. Dennoch ist die Studie nach unseren Recherchen die bisher größte Befragung von Spitzensportlern zum Thema Dopingberichterstattung in Deutschland. Positiv werden die Verteilungen nach Geschlecht, Alter und Bildungsgrad gesehen. Die Teilnahme von Athleten aus 30 verschiedenen olympischen Sportarten bietet zudem eine hohe Sportarten-Varianz, wobei Sportarten, die häufiger zum Gegenstand der Dopingberichterstattung werden, genauso dabei sind wie Sportarten, die selten oder nie Gegenstand der Dopingberichterstattung sind. Nachteilig ist, dass insgesamt nur 186 Athleten ihren Sportverband, dem sie angehören, angeben wollten. Und dass viele Sportarten nur durch einen oder 2 Sportler vertreten sind, lässt sportartenspezifische Auswertungen nicht sinnvoll erscheinen.

## Fazit

Die Ergebnisse zu Wahrnehmung und Bewertung der Dopingberichterstattung zeigen, dass viele Athleten die Dopingberichterstattung für wichtig und notwendig erachten, dass sie aber die Art und Weise, wie Medien über Doping berichten, durchaus kritisch sehen. Unabhängig davon meinen fast alle Athleten, dass Dopingberichte weder ihre mentale und körperliche Leistungsfähigkeit noch ihr Verhalten im Training und Wettkampf beeinflussen können. Ob tatsächlich so viele Spitzensportler „immun“ gegenüber der Dopingberichterstattung sind, wie sie vorgeben zu sein, muss hier offenbleiben.

Im Ansatz der „reziproken“ Effekte wird zwischen Wahrnehmungs- und Wirkungsdimensionen unterschieden. Diese Studie liefert erste Hinweise darauf, dass durch Dopingberichterstattung Effekte bei den Dimensionen „Selbstwahrnehmung“ und der „Berichtswahrnehmung“ auftreten. Im Gegensatz dazu zeigen sich „Wirkungserfahrungen“ bzgl. Training und Wettkampf mehrheitlich nicht. Bezüglich der „Wirkungsdimensionen“ lassen sich Einflüsse auf der kognitiven und partiell auch auf der emotionalen Ebene erkennen, aber nur ganz selten auf der konativen Ebene.

## Supplementary Information

Onlinematerial: Stichprobencharakteristika

## Data Availability

Die während der vorliegenden Studie erzeugten und/oder analysierten Datensätze sind auf begründete Anfrage bei der Korrespondenzperson erhältlich.
