# Supplementary material for: Media coverage of doping: subjective perceptions, evaluations, and perceived effects among elite athletes
Source: Bundesgesundheitsblatt Gesundheitsforschung Gesundheitsschutz. 2026 Jun 2;69(7):750–8. [Article in German] doi: 10.1007/s00103-026-04250-6 (PMC13323818; doi:10.1007/s00103-026-04250-6)
Supplement: Supplementary file 1 — Onlinematerial: Stichprobencharakteristika [file 103_2026_4250_MOESM1_ESM.pdf]

Onlinematerial: Stichprobencharakteristika

Tab. Z 1 zu Alter, Geschlecht, Bildung (Angaben in Prozent und als Anzahl (n))

		Geschlecht (n=219)			Bildung (n=215)				
		Gesamt	Männ-lich	Weib-lich	Promo-tion	Stu-dium	Fach-abitur / Abitur	Berufs-schule / Lehre	Haupt-/ Realschule
		100%	48,4	51,6	1,9	33	50,7	8,4	6
Altersgruppen (n=214)	18-20-Jährige	21,9 (n=47)	46,8 (n=22)	53,2 (n=25)		2,2 (n=1)		80,0 (n=36)	17,8 (n=8)
	21–23-Jährige	19,2 (n=41)	47,5 (n=19)	52,5 (n=21)	2,4 (n=1)	22,0 (n=9)	61,0 (n=25)	9,7 (n=4)	4,9 (n=2)
	24–26-Jährige	20,6 (n=44)	40,9 (n=18)	59,1 (n=26)	2,3 (n=1)	32,6 (n=14)	55,8 (n=24)	9,3 (n=4)	
	27–29-Jährige	19,1 (n=41)	41,5 (n=17)	58,5 (n=24)		75,0 (n=30)	20,0 (n=8)	5,0 (n=2)	
	30–35-Jährige	13,5 (n=29)	72,4 (n=21)	27,6 (n=8)	3,4 (n=1)	41,4 (n=12)	27,6 (n=8)	20,7 (n=6)	6,9 (n=2)
	36–40-Jährige	2,9 (n=6)	66,7 (n=4)	33,3 (n=2)		16,7 (n=1)	50,0 (n=3)	33,3 (n=2)	
	Über 41-Jährige	2,8 (n=6)	33,3 (n=2)	66,7 (n=4)	16,7 (n=1)	33,3 (n=24)	33,3 (n=2)		16,7 (n=1)
	Gesamt [n]	214	103	110	4	69	70	50	13

Tab. Z 2 zur Spitzenverbandszuordnung und Einteilung nach Doping-Risikogruppierung laut NADA (Angaben in Prozent und als Anzahl (n))

**Teilnehmer nach Spitzenverbandszuordnung (n=186)**		
Risikogruppe A	*Risikogruppe B*	*Risikogruppe C*
Skisport (20,4%; n=38)	Ringen (4,8%; n=9)	Schieß- und Bogensport (8,1%; n=15)
Leichtathletik (12,4%; n=23)	Hockey (4,3%; n=8)	Segeln (2,2%; n=4)
Bob- und Schlittensport (6,5%; n=12)	Judo (3,2%; n=6)	Curling (1,6%; n=3)
Radsport (6,5%; n=12)	Volleyball (2,7%; n=5)	Eiskunstlauf (1,6%; n=3)
Rudern (4,3%; n=8)	Eishockey (2,2%; n=4)	Taekwondo (1,6%; n=3)
Schwimmen (2,7%; n=5)	Basketball (2,2%; n=4)	Moderner Fünfkampf (1,1%; n=2)
Kanu (2,7%; n=5)	Tennis (1,1%; n=2)	Alpenverein/Bergsport (0,5%; n=1)
Eisschnelllauf / Shorttrack (2,2%; n=4)	Fechten (0,5%; n=1)	Badminton (0,5%; n=1)
Turnen (1,6%; n=3)	Fußball (0,5%; n=1)	Snowboard (0,5%; n=1)
Gewichtheben (0,5%; n=1)	Handball (0,5%; n=1)	Wellenreiten (0,5%; n=1)
**111**	**41**	**34**
